# Supplementary material for: Are carbon emissions trading and green financial instruments synergistic? -Comprehensive quantitative research based on content analysis
Source: PLoS One. 2024 Mar 7;19(3):e0298601. doi: 10.1371/journal.pone.0298601 (PMC10919601; doi:10.1371/journal.pone.0298601)
Supplement: S1 Data — (ZIP) [file pone.0298601.s001.zip › Data/地级市Z_F工作报告.zip!/╣π╬≈/╬α╓▌╩╨/└┤╫╘╣1⁄2╓┌║┼í╛┬φ┐╦╩2╛▌═°í┐_2015.docx]

各位代表：

　　现在，我代表市人民政府向大会作政府工作报告，请予审议，并请市政协各位委员提出意见。

一、2014年工作回顾

过去的一年，经济形势比预料更为严峻，困难矛盾超出预期。面对经济下行的巨大压力，我们在市委的坚强领导下，认真贯彻落实中央和自治区的决策部署，围绕市十三届人大五次会议确定的目标任务，迎难而上，主动作为，奋力攻坚，以“三年一工程 ”为抓手，全力以赴稳增长、调结构、促改革、惠民生，全市经济社会发展总体平稳，实现了稳中提质、稳中增效、稳中有进。

同时，我们还推动促成了一些事关全局和长远发展的大事、要事：

国家批复《珠江—西江经济带发展规划》，粤桂合作特别试验区建设被纳入规划上升到国家战略层面，粤桂两省（区）联合全面启动试验区建设；

梧州至南宁、广州、深圳等城市开通高铁，迈入高铁时代；

梧州西江机场迁建获国家批复立项；

微软公司、华润集团、中钢集团、国光集团等国际知名企业落户梧州；

西江经济带基础设施建设大会战和城市建设三年提升工程全面启动；

梧州被列为全国首批四个工业用地市场化配置改革试点城市之一，等等。

一年来，我们主要做了以下工作：

　　（一）综合施策稳增长，经济保持平稳运行。积极应对复杂严峻经济形势，认真贯彻落实中央和自治区稳增长决策部署，出台40条稳增长措施，有效发挥投资和工业对稳增长的支撑作用。

持续打好项目攻坚战，实行市领导联系重大项目（事项）制度，全市安排重点项目1057个，完成投资827亿元，占全市固定资产投资的95%。

实施新常态下工业巩固提升工程，帮助企业解难题、上规模、提效益，规模以上工业企业实现利润增长34.8%，全市110家重点工业企业有80%以上实现增长，成为稳增长的主力军。

大力发展非公有制经济，推出30条扶持措施，全市非公企业总量扩大到1.8万多户，非公经济增加值占全市地区生产总值近70%。

积极防范和处置涉企金融风险，维护了良好的金融生态环境，金融对实体经济的支持力度不断加大，全市金融机构各项贷款余额达622.3亿元，增长15.2%。

多举措扩大消费，全年实现社会消费品零售总额330亿元，增长13%。

（二）转变方式调结构，发展质量不断提升。加快传统产业转型升级，完成更新改造投资380.6亿元。积极发展循环经济和战略性新兴产业，再生资源和再生不锈钢制品两大产业实现总产值850亿元，电子信息、建材等产业总产值增长20%以上。支持企业技术创新，新增3家自治区级企业技术中心和4家自治区级研发中心。大力发展农业优势特色产业，粮食生产实现稳产丰收，“菜篮子”基地面积达1.2万亩，水果、六堡茶和中药材种植总面积达100万亩，规模养殖场达839个，岑溪市西江果蔬产业（核心）示范区被授予“广西现代特色农业（核心）示范区 ”称号。积极培育发展新型农业经营主体，新增各类农民专业合作社145个，新发展家庭农场98个，新增农业产业化重点龙头企业20家，农业基础地位进一步巩固。加快发展商贸物流业，市商贸物流园区、毅德商贸物流城、中恒国际医药物流产业园、岑溪泽仁现代物流园等项目加力推进，旺城广场、梅西生活广场等建成营业。抓住高铁开通机遇推动旅游业发展，新增国家3A级旅游景区2家、自治区四星级乡村旅游区3家和四星级农家乐5家，蒙山长坪乡获评为全国首批少数民族特色村寨，岑溪市归义镇、南渡镇吉太三江口村和藤县象棋镇道家村获“广西特色名镇名村”称号。

（三）立足长远固基础，发展后劲持续增强。加快工业园区基础设施和配套设施建设，推动产城融合发展，完成园区基础设施投资25.8亿元。

全力推进交通基础设施建设，柳肇铁路柳梧段获批立项，赤水圩作业区进港铁路全线动工，洛湛铁路扩能改造工程前期工作加快推进；梧州至贵港高速公路主体工程完工，岑溪至水汶高速公路加快推进，梧州至柳州高速公路、市环城高速公路加快建设，荔浦经蒙山至玉林高速公路、梧州至信都高速公路开展前期工作；长洲水利枢纽三线船闸建成通航，四线船闸加紧收尾工作，一批重点港口码头项目顺利推进；龙圩至藤县潭东一级公路路基工程完工，路面施工加速推进；全市863个建制村通硬化路率达100%。

加快推进信息通信基础设施建设，实施微软创新中心、中兴云数据中心等信息化项目，启动信息惠民国家试点城市建设。

大力实施民生水利工程，完成维修加固水库40座、堤防护岸26公里，新建农村饮水安全工程114处，全年完成水利项目投资15.8亿元。

（四）大力创新促改革，开放合作水平显著提高。稳步推进行政审批制度改革，取消、下放和调整行政审批项目265项。大力推进农村综合改革，农村金融改革、土地确权登记发证试点、“小块并大块”等改革工作有序推进。工商登记制度改革取得实效，新登记的有限责任公司和注册资本金分别增长163%和350%。全面落实珠西规划，勇作向东开放“排头兵”，当好改革创新“试验田”。深化与肇庆、云浮的战略合作，与佛山签署战略合作框架协议，与广州达成战略合作意向。推动粤桂合作特别试验区体制机制创新，粤桂两省（区）建立了联席会议制度，出台了实施方案；试验区与多家金融、股权和证券机构签订了战略合作协议，首批16家企业在广东金融高新区股权交易中心挂牌，广西17家银行业金融机构联合支持试验区建设；探索“园中园”开发模式，新加坡产业园、国光电子产业园、中节能环保产业园等项目进驻试验区。大力发展会展经济，成功举办第十一届梧州宝石节、第十届中国茶叶经济年会暨六堡茶博览交易会、第四届广西发明创造成果展览交易会和首届中国（梧州）国际健康养生产业资源合作与投资促进大会。承接产业转移保持良好态势，完成招商引资区外到位资金491.5亿元，增长9.9%。

（五）优化布局拓空间，生态宜居城市建设取得新进展。开展城市总体规划修编和11项专项规划编制，明确未来20年城市发展蓝图。

大力实施城市建设三年提升工程，安排建设项目150多项，预计总投资约460亿元。

加快城市新区开发建设，红岭新区中央商务区、玫瑰湖景观提升工程、太和新城等项目加快实施，公共配套设施功能日趋完善；苍海新区环湖道路、跨湖大桥、岸线景观、环城水系等项目稳步推进，第六届广西园博会展园动工建设。

蒙山县获得“广西园林城市”称号。

实施棚户区改造，已启动14000多户改造工作。

完成了6920户农村危房改造任务。

持续加强生态文明建设，全市森林覆盖率达75.8%，环境空气质量优良率为99.7%，江河水质达标率保持100%。

清洁乡村活动实现全覆盖，生态乡村活动全面启动，建成乡镇生活垃圾中转站19个、乡村垃圾处理设施824个，农村生产生活环境有了较大改善。

（六）加大投入惠民生，社会各项事业协调发展。公共财政支出更多地向基层和民生倾斜，民生支出占比达到79%。12项为民办实事工程全面完成，总投资超过49亿元。精准扶贫取得实效，实现6万贫困人口脱贫。多举措促进城乡居民增收，城镇居民人均可支配收入增长7.7%、农民人均纯收入增长11.6%。着力推进教育发展十大重点工程，促进各类教育均衡发展，改建农村幼儿园项目135个，资助贫困学生24万多人次，1.7万名进城务工人员随迁子女全部安排就近入学，11.4万名义务教育阶段留守儿童全部正常就学。策划开展“圆梦1+1”爱心助学公益活动，全市858个乡村教学点受益。加大社会保障工作力度，80岁以上高龄老年人生活补贴政策得到有效落实，企业退休人员养老待遇比上年提高10.2%，城镇居民医保补助标准比上年提高14.3%，城乡居民社会养老保险实际参保人数达120万人以上，被征地农民参加养老保险人数达1.46万人。城市低保、农村低保月人均补助水平分别提高到260元和102元。持续推进“安居乐业”工程，开工建设保障性住房11536套，基本建成6144套；全市城乡新增就业9.49万人次，城镇登记失业率为3.2%，低于目标控制数1.3个百分点。加大文化惠民力度，建成村级公共服务中心68个、村村通乡镇无线发射台站6个，超额完成自治区下达的1万多场农村公益电影放映任务。全力做好广西第十三届运动会的筹备工作，综合馆和游泳馆主体施工按计划推进，红岭体育场主体工程完工。医药卫生体制改革初见成效，新农合“一卡通”惠及264.3万农民，83%以上的村卫生室实现即时刷卡结算。稳步推进人口和计划生育工作，“单独两孩”政策全面落实。狠抓安全生产不放松，事故起数、直接经济损失等指标均比上年下降。开展食品药品专项整治，解决了一批群众反映强烈的食品药品安全问题。启动实施质量强梧战略，颁发首届市长质量奖。深入开展“法律六进”和“大接访、大排查、大调解”活动，信访总量呈下降趋势，矛盾纠纷调解成功率达97%。继续完善社会治安防控运行机制，行政村驻村和挂点民警配备率达100%，开展打击传销集群战获得公安部肯定。扎实推进平安梧州建设，建成政府应急指挥中心，圆满完成重大节会期间维稳安保任务，社会保持和谐稳定。

（七）为民务实转作风，政府自身建设扎实推进。深入开展党的群众路线教育实践活动，严格落实中央“八项规定”和国务院“约法三章”，认真整改“四风”方面存在的突出问题，停止新建政府性楼堂馆所，严格控制财政供养人数，市本级公共财政安排的“三公”经费支出下降了6%，文件数量、工作会议、评比达标活动分别压缩了16.7%、17.1%和41.1%。加大行政监察和审计监督力度，政府廉政建设有力推进。加快建设法治政府，抓好依法行政示范点建设，抓好重大项目法律顾问服务工作，将依法行政工作纳入绩效考核。加大政务公开力度，健全政府信息发布制度，更好地保障了人民群众的知情权、参与权和监督权。自觉接受人大及其常委会法律监督和工作监督，主动接受政协民主监督，人大代表建议和政协提案办结率100%。

一年来，国防教育和后备力量建设扎实推进，全民国防意识不断加强，双拥共建工作取得新成绩。中直、区直驻梧各单位为全市经济社会发展作出了突出贡献。编制、民族、宗教、外事侨务、对台、统计、物价、国资、供销、农机、地震、人防、移民等工作取得了新进展。工会、共青团、妇女儿童、残联、老龄、科协、社会科学、慈善、档案、地方志等工作取得新进步。

各位代表，过去的一年，在困难和挑战面前，全市上下顽强拼搏、共克时艰，积极适应经济增速换档新常态，始终坚持用最大的努力来争取最好的结果，取得的成绩来之不易。这是自治区党委、政府正确领导的结果，是市委统筹把握、科学决策的结果，是市人大依法监督和市政协民主监督的结果，是全市人民万众一心、奋力拼搏的结果。在此，我代表市人民政府，向全市各条战线上的干部群众，向人大代表、政协委员，向各民主党派、工商联、无党派人士、人民团体，向离退休老领导、老同志，向驻梧部队、武警官兵、公安干警，向所有关心支持梧州发展的社会各界人士，表示崇高的敬意和衷心的感谢！

我们也清醒地看到，我市发展中还面临一些较大的困难和严峻的挑战：因宏观经济趋紧，发展速度总体放缓，2014年部分经济指标未能达到年初预期；项目推进困难加大，土地、资金等要素“瓶颈”制约仍然突出；结构性矛盾逐渐显现，转型发展任务艰巨；财政收支矛盾加大，改善民生仍有差距；政府职能有待加快转变，发展环境有待不断优化，等等。对此，我们一定以高度负责的态度，积极应对，主动作为，认真解决。

二、2015年工作总体要求和目标任务

今年是全面深化改革的关键之年，是全面贯彻依法治国方略的开局之年，也是全面完成“十二五”规划的收官之年。当前我国经济发展步入新常态，经济由高速增长转向中高速增长。从表象看这是增长速度的换档放缓，但本质上是结构转型、方式转变、动力转换和体制转轨，其核心就是经济发展方式从规模速度粗放型增长转向质量效益集约型增长。我市还处在工业化中期阶段，经济规模不大，产业层次不高，发展不平衡、不协调等固有矛盾在新常态下将会表现得更加突出，面临着加快做大总量和推进转型升级的双重压力，保持经济增长的合理区间与推进结构的战略性调整任务紧迫而艰巨，经济持续下行的态势和发展环境仍然非常严峻。但总的来看，我市仍处于大有可为的战略机遇期：珠江—西江经济带建设上升为国家战略，两广签署粤桂合作共同行动计划、推进高铁经济带规划建设，自治区深入实施“双核驱动”战略，开展西江经济带基础设施大会战，支持粤桂合作特别试验区先行先试，等等。同时，经过多年努力，我市地区生产总值突破1000亿元大关，进入跨越发展新阶段。我们要抢抓机遇，开拓进取，主动作为，克难攻坚，不断提高新常态下推动发展的能力和水平。

今年政府工作的总体要求是：深入贯彻党的十八大、十八届三中、四中全会和习近平总书记系列重要讲话精神，坚持稳中求进的工作总基调，坚持以提高经济发展质量和效益为中心，紧紧围绕加快建设西江黄金水道上向东开放的龙头城市和全面建成小康社会的奋斗目标，认真落实珠西规划和自治区“双核驱动”战略部署，积极对接粤港澳，主动适应新常态，把转方式调结构放到更加重要位置，突出创新驱动，全面深化改革，扩大开放合作，继续实施“三年一工程”，加快建设“两区一城”，切实保障和改善民生，促进经济平稳健康发展与社会和谐稳定。

经济社会发展的主要预期目标是：地区生产总值增长8.2%，规模以上工业增加值增长10%，财政收入增长5%，固定资产投资增长12%，社会消费品零售总额增长12%，进出口总额增长10%，万元生产总值能耗下降2%，主要污染物排放总量控制在自治区下达的指标之内，居民消费价格涨幅控制在3.5%以内，城镇居民人均可支配收入增长8.5%，农民人均纯收入增长11%，城镇登记失业率控制在4.5%以内，人口自然增长率控制在11‰以内。

确定以上目标，主要是立足于我市经济社会发展的阶段性特征，加快完成工业化中期任务，既与“十二五”规划发展目标相衔接，又充分考虑了到2020年全面建成小康社会的要求；既积极有为、加油鼓劲，又实事求是、务求实效；既顺应新常态的要求，体现质量和效益并重、生态文明和民生福祉并举，又为转型升级赢得时间和空间，为保障和改善民生创造必要条件。在实际工作中，我们将坚持稳中求进、好中求快，能快就不要慢，尤其是针对传统产业、优势产业要重点推动提质增效，而对基础较好的若干战略性新兴产业、高新技术产业，则力求赶超跨越。

三、2015年主要工作安排

新常态要有新担当、新作为。为确保实现今年各项目标任务，要突出抓好以下几方面工作：

（一）深化改革创新，着力推动开放合作形成新格局。

改革就是增强活力，开放就是增强聚合力。要通过新一轮的改革创新和开放合作，激发市场和社会活力，推进经济社会新发展。

深化重点领域改革。加大行政审批制度改革力度，规范行政审批管理，完善行政审批事项目录管理，进一步做好“接、放、管”，全面实施工商登记“先照后证”，为基层、企业和各种市场主体继续松绑放权。深化投融资体制改革，扩大民间资本市场进入范围，降低准入门槛，鼓励和支持国有企业、国有控股企业充分运用金融产品，利用信托、融资租赁等方式，社会资本以合资、独资、特许经营等方式，参与项目建设运营。抓好国家工业用地市场化配置改革试点城市工作，用好、用足、用活各种政策，探索健全工业用地多种供应形式、多主体供应工业用地市场流转体系和价格体系。加强财政管理改革，全面落实新预算法，强化预算支出约束；推广预算绩效管理，推行政府购买服务，推进公务用车制度改革。深化国资国企改革，推进政资政企分开。

扩大对外开放合作。坚持“东向”战略，深化与佛山、肇庆、云浮的战略合作，建立政府、民间、企业多层面交流合作机制，实施肇梧合作2015年重点工作计划，建立梧云、梧佛市长联席会议制度，推动与广州签订战略合作协议，促进粤桂合作特别试验区与深圳前海深港合作区开展务实合作。

积极实施珠西规划共同行动计划，积极衔接国家“一带一路”战略和两广高铁经济带规划，加速与珠三角在规划、产业、基础设施、体制、市场和要素等方面对接，打造两广经济一体化先行区。

加大招商引资力度，围绕循环经济、电子信息、生物医药、新能源汽车、健康养生、电子商务等行业领域，包装策划一批投资合作项目，持续开展“央企入梧”、“民企入梧”和“银企入梧”等专题招商活动，大招商、招大商，实现招商引资质优量增。强化招商项目跟踪服务，狠抓签约项目落地开工，力争全市招商引资区外到位资金达535亿元以上。

加强东西部金融合作，加快引进全国性股份制银行到梧设立分支机构。

着力提升贸易便利化水平，大力支持加工贸易发展，继续推动梧州口岸“三个一”通关改革和梧州港扩大开放，实现外贸进出口总值13.2亿美元以上。

进一步加强教育、文化、体育、卫生等社会事业对外交流合作，提升发展水平。

推进粤桂合作特别试验区先行先试。全面落实试验区建设实施方案和肇梧两市政府合作协议，争取两省（区）出台试验区改革创新总体方案和管理办法，推动成立试验区联合管委会，力争在重点领域和关键环节取得重大突破。同步推进起步区、先行区和拓展区建设，推动塘源一路一期、塘源污水处理厂、公共服务中心等一批重点基础设施项目建成使用，推动航天北斗超电容、粤创高科裸眼3D等一批重大项目实质性开工。支持国内外知名企业在试验区开展“园中园”建设，加快推进国光电子产业园、新加坡产业园等项目。探索融资模式多元化和开发运作市场化，着力建设粤桂合作金融安全区，推动试验区金融创新先行先试。落实国家及两省（区）优惠政策，加快申报设立梧州综合保税区，着力推进试验区加工贸易园区、生产性服务业示范区、东西部金融合作示范区建设，打造中国—东盟环保技术和产业合作交流示范基地、粤桂合作企业总部基地和创业创新基地。力争试验区全年实现规模以上工业总产值增长26%以上，招商引资实际到位资金30亿元以上。

（二）加大投资力度，着力推动项目建设实现新突破。

稳增长关键是稳投资，要以重大项目为支撑，加大投资力度，突出扩量提质保进度，持续掀起抓项目、上项目、建项目的热潮，进一步增强投资的拉动力。

强化重大项目带动作用。努力开辟投资新领域，扩大有效投资，优化投资结构，鼓励引导资金特别是社会资本投向重大基础设施、重点产业园区、战略性新兴产业、现代服务业、城镇化建设、社会事业、生态环保等领域。拟安排投资超亿元的重大项目460项，总投资3164.6亿元，年度计划投资407.4亿元。其中，续建项目247项，年度完成投资276.6亿元；计划新开工项目122项，年度完成投资130.8亿元；前期项目91项。全年计划完工项目105项，年度完成投资113亿元。

　　加强重大基础设施建设。以实施西江经济带基础设施建设大会战为抓手，着力推动交通、能源等领域重大基础设施建设，进一步增强发展的支撑能力。公路方面，确保梧州至贵港高速公路和龙圩至藤县潭东一级公路建成通车，以及岑溪至水汶高速公路主体完工，加快市环城高速公路、梧州至柳州高速公路梧州段、207国道东绕城过境一级公路等项目建设，争取新开工荔浦至玉林高速公路梧州段、龙圩经大坡至新地二级公路，积极推进梧州至信都高速公路、梧州至藤县藤州镇公路等项目前期工作。

　　铁路方面，争取柳肇铁路梧州段、洛湛铁路永州至玉林段扩能项目早日开工建设，推动岑罗铁路复工建设，建成梧州港赤水圩作业区码头进港铁路，扎实推进柳韶铁路梧州段、洛湛铁路苍梧（石桥北）站扩建等项目前期工作。

　　水运方面，重点推进桂江航道整治、大利口码头、塘源综合码头等项目建设。

　　航空方面，开工建设梧州西江机场，力争2017年建成投用。

　　防洪方面，加快建设长洲南北堤、高旺防洪堤、平浪防洪堤、塘源防洪堤、藤县河东防洪堤，开工建设三龙防洪堤。

　　能源方面，力促旺村水利枢纽竣工投产，大力推进城镇天然气应用，进一步优化电网布局。

　　加强项目管理和要素保障。围绕国家、自治区重点建设方向及资金信贷投向，加大项目研发生成力度，储备一批好项目大项目。安排项目前期工作经费，对条件成熟的项目优先安排经费，优先保障土地资金需求。落实项目推进责任机制，细化项目“形象进度”计划，破解用地、资金、土地征拆等瓶颈制约因素。加强工程招投标管理，强化项目资金的运行监督和跟踪审计，确保每个项目都建成“阳光工程”、“廉洁工程”。

（三）突出工业提质，着力推动转型升级迈出新步伐。

工业是经济增长的主引擎，也是转方式调结构的主战场，要把工业提质增效放在更加突出的位置。

推动产业升级。加快发展千亿元产业，促进国龙15万吨再生塑料、盈锦化工机械、鑫华通、中晖电子等重点项目建成投产，推动中钢工业园、安富利电子产业园等重点产业基地建设，不断完善再生资源、再生不锈钢制品、电子信息产业链条。

改造提升传统优势产业，鼓励和支持食品医药、陶瓷、钛白粉、林产林化等产业“二次创业”，抓好志冠胶原蛋白等256项工业技改项目，力争完成更新改造投资420亿元，增长12%以上。

培育发展战略性新兴产业，推动中节能环保产业园等节能环保产业项目、重型起重机械等先进装备制造项目开工建设，促进高性能钕铁硼永磁材料等新材料项目建成投产，推进微软创新中心、阿里巴巴·梧州产业带、中兴云数据中心等新一代信息技术项目建设，不断推动产业向价值链高端演进。

实施创新驱动。加快构建以企业为主体、市场为导向、产学研协同的科技创新体系，推动工业发展从要素驱动、投资驱动向创新驱动转型。鼓励和支持企业进行技术、产品、管理、业态、市场等方面创新，引导创新要素向企业集聚，提升创新能级和创新效率。推进工业创新发展，培育国家级高新技术企业2家以上，建设市级工程技术研究中心5个，支持梧州高新区申报国家级高新技术开发区，加快再生不锈钢制品研发机构和科技企业孵化基地建设。深入实施质量强梧战略，筹建西江流域质量技术检测研究中心，推动工业企业质量品牌和知识产权优势企业建设，扩大老字号品牌的影响力。实施发明专利倍增计划，力争申请发明专利达850项。落实小微企业扶持政策，为创业创新营造良好环境，推动大众创业、万众创新。

做强园区经济。以培植百亿元园区、千亿元园区为目标，大力推动园区提质扩容，全面加快园区建设进程。梧州工业园区围绕提升综合竞争力，抓好中恒、神冠、住电汽车线束等重点企业和项目，扎实推进赤水港临港经济区和电子信息产业园区建设。

进口再生资源加工园区围绕做足上下游产业链条，促进骆驼蓄电池、嘉禾汽车拆解和再制造等深加工项目建成投产，启动二期开发建设。

不锈钢制品产业园区围绕产业向中高端发展，推进金海冷轧薄板、正业不锈钢等项目建设，加快窝田集中区基础设施建设。

工业新城围绕粤桂合作，加强产城互动，推进道路、给排水、电力等基础设施项目，加快工业企业落户步伐。

陶瓷产业园区围绕循环化改造，推进碳歌陶瓷等一批重点企业改造升级。

统筹抓好苍梧县旺甫工业小镇、岑溪西部农民创业园、蒙山茧丝绸产业园区等县域工业集中区建设。力争全年园区完成基础设施投资29亿元，实现工业总产值1900亿元。

壮大企业实力。持续开展“服务企业年”活动，健全企业信息服务体系，建立企业运行监测预警机制，及时发现和帮助企业解决实际困难和问题，促进企业达产达效。加快推进中小企业信用体系建设，大力破解中小企业融资难题，做好“助保贷”的扩面增量工作，力争“助保贷”新增贷款2亿元，业务范围覆盖全市，受惠企业面扩展到全部工业企业。加大企业培育力度，扶持传统优势企业做大做强，形成大企业支撑大产业的格局，力争新增规上企业25家，新增亿元企业4家，其中超10亿元的企业2家，超100亿元的企业1家。

（四）围绕农业提效，着力推动农业发展走出新路子。

在稳定粮食生产和主要农产品供给的前提下，加快转变农业发展方式，持续增加农民收入，走生产高效、产品安全、资源节约、环境友好的现代农业之路。

壮大优势特色农业。严守耕地红线，粮食播种面积稳定在237万亩以上，粮食总产82万吨。

继续抓好优势特色产业，确保水果、六堡茶和中药材种植总面积稳定在100万亩以上，蔬菜复种面积扩大到100万亩。

深入实施“绿满八桂”造林绿化工程，全年植树造林20.5万亩以上。

加快水产畜牧业转型升级，重点发展特色高效生态养殖业，大力推进生猪、三黄鸡、蜂、草食动物等产业标准化发展，积极发展黑土猪、娃娃鱼等特种养殖业，稳定全市肉类、水产品总产量。

突出抓好梧州（岑溪）现代特色农业核心示范区建设，逐步实现每个县（区）都建成一个自治区级或市级示范区。

加强农民职业培训，积极发展农业社会化服务，落实国家强农惠农富农政策，做好良种补贴、农业保险等财政补贴工作，多渠道促进农民增收。

推进农业专业化生产和产业化经营。全面落实农村耕地所有权、承包权和经营权三权分置，继续抓好农村土地承包经营权确权登记颁证工作，扩大试点范围。积极引导农村土地经营权有序流转，稳步推进农业适度规模经营，培育发展农业龙头企业、专业大户、农民专业合作社、家庭农场等新型农业经营主体，力争新增龙头企业5家以上，新增新型农业合作组织50家以上。支持农产品加工和流通，建立特色农产品交易市场，鼓励发展农产品电子商务，促进主要农产品顺产顺销。拓展农业新业态，促进农资流通、科技服务、创意农业等农业服务业发展。推进农业机械化，力争全市耕种收综合机械化水平达到42%以上。开展特色农业产业“品种品质品牌”提升行动，抓好农产品标准化生产和质量安全，努力打造西江经济带生态农产品基地，确保全市蔬菜、水果、茶叶抽样产品例行检测合格率达96%以上。

推进农村基础设施建设。筹措资金13.5亿元，基本完成病险水库除险加固工作，继续抓好中小河流治理、农田水利、农网改造、河道整治、水土保持等项目建设。持续完善农村公路路网，在实现行政村100%通硬化路的基础上，启动一批县乡公路联网、连通公路、安保工程、危桥改造等项目的建设，努力提升农村交通综合服务能力。

（五）提速发展三产，着力推动结构调整取得新成效。

壮大第三产业是优化经济结构的主攻方向，要优先发展生产性服务业，加快发展现代服务业，努力提升第三产业增加值占比。

加快发展现代物流业。实施“大港口、大通道、大物流”发展战略，加强粤桂互联互通，打造节点物流，建设区域性物流集聚区、货运集散中心。以服务我市优势产业为重点，分门别类规划建设产品生产、加工、集散基地，形成门类齐全、特色鲜明的专业配送市场。大力推进市商贸物流园区等重点物流园区建设。积极发展第三方物流，鼓励和引导大型集团公司剥离自营物流服务功能，逐步发展成为独立的物流企业，大力培育社会化、专业化的物流龙头。

加快发展商贸流通业。按照现代商品市场体系要求，调整市场结构，优化商贸业网点布局。

大力引进国内外知名连锁百货店，建设主题特色鲜明、综合配套齐全的大型购物中心。

引导大型综合超市向社区拓展，鼓励标准超市向精品超市、生鲜超市转型，支持专卖店向品牌旗舰店、体验店等方式发展，大力发展城市综合体等新型业态。

合理布局商业中心，重点建设河东老城区商业圈、苍海新区商业圈、红岭商务区、新加坡印象、旺城广场商圈等精品特色商业街区，加快构建具有梧州特色、影响周边、辐射两广的新型商贸流通体系。

加快发展休闲旅游、健康养生业。完善旅游基础设施，着力打造龙头景区，重点推进苍海生态休闲旅游度假胜地建设，支持石表山景区创建国家级生态旅游示范区，加快思良江休闲农业观光带建设，积极申报国家级湿地公园。

扎实推进特色旅游名县名镇创建工作，完善激励创建特色旅游名县名镇的支持政策，继续抓好一批乡村旅游区和农家乐项目。

推进县域旅游与全市重大旅游品牌的联合推介、捆绑营销，促进区域旅游跨越发展。

大力开展高铁旅游联合营销，全面融入泛珠三角旅游圈、大桂林旅游圈，开拓旅游客源市场，争取全市接待旅游人数达1500万人次，增长15%；旅游总收入145亿元，增长20%。

坚持高端化、特色化、规模化和国际化发展方向，积极推进岭南生态养生城建设，抓好“全国老年友好型城市”试点和“中国长寿之乡”申报工作，打造粤港澳“后花园”。

强化政策保障。设立引导资金，出台扶持政策，激励和引导第三产业发展，重点支持大型物流企业发展，支持产业园区建设物流平台，支持民间资本参与公共服务领域建设，支持推动商贸企业“个转企”、“小上限”等，努力营造第三产业发展良好环境。鼓励支持电子商务、商务会展、文化创意等新业态加快发展，着力优化三产内部结构。落实国家和自治区服务业价格、财税、土地等优惠政策，积极争取我市更多企业和项目列入自治区服务业“三个一百”工程。

（六）加快新型城镇化，着力推动城乡建设展现新面貌。

新常态下，城镇化是最大的内需，要用好这一强大引擎，全面落实新型城镇化实施方案，构建城乡一体发展新格局。

　　实施城市建设三年提升工程。围绕城市新区拓展、旧城改造、城市道路及基础设施建设、城市公园及绿化美化建设、城市环境综合整治等7大任务，重点实施138个项目，确保年度完成投资63.4亿元。

突出抓好苍海新区建设，加快推进东环大道、望江交通隧道、东部山体林相改造等道路及景观工程，高质量完成园博园建设任务，承办一届“永不落幕”的广西园博会。

加快推进棚户区改造和城中村改造，重点抓好龙平片区、百花冲片区、富民片区等棚户区改造项目，开工建设棚户区改造住房22297户。

加快推进西江三桥、四桥建设，统筹开展城市给水、排水、污水等管线改造及建设，完成新兴二路、新兴三路、东湖路等一批市政道路改造。

建成红岭体育场、综合馆、游泳馆、网球中心等区运会比赛场馆。

完成《梧州市龙圩区城市控规》、《梧州市水网专项规划》等11个专项规划编制任务。

大力开展市容环境综合整治，优化城市出入口道路，查处占道经营、私搭乱建、不遵守交通规则等违规行为，创造和谐舒适的人居环境。

加强城市无障碍设施建设，全力创建全国无障碍城市。

完善城镇体系。加快实施大县城战略，大力推动藤县撤县设市，推进岑溪新型城镇化试点县建设，支持蒙山县打造桂东精品小城市，全面加快苍梧新县城的建设步伐。大力推动小城镇建设，突出抓好苍梧旺甫、岑溪归义、藤县太平、蒙山新圩等重点镇的建设，打造一批经济强镇、旅游名镇、特色小镇，把小城镇建设成为服务“三农”、促进农民就地城镇化的重要载体。着力推动产城融合，适度调整梧州工业园区、再生资源加工园区等产业园区的用地结构和功能布局，建立各种公共设施和服务平台，推动城镇与园区同步建设、整体推进。围绕城乡发展一体化，扎实推进新农村综合示范样板点建设。

　　推进农业转移人口市民化。深化户籍制度改革，创新人口管理，逐步建立以合法稳定就业和合法稳定住所为户口迁移条件、以经常居住地登记户口为基本形式的人口登记制度，打破农业户口和非农户口的二元性质划分。全面开放建制镇落户限制，有序放宽中心城区落户条件，鼓励农民工返乡创业，坚持自主意愿、因地制宜的原则，引导农业转移人口就地市民化，促进人口有序流动、合理分布。逐步消除城乡户籍壁垒，探索进城落户农民保留土地用益物权，使农业转移人口进得来、留得下、过得好、有保障、有尊严。

　　推进公共服务均等化。坚持以人为本，保障农业转移人口在劳动就业、子女入学、住房保障、社会福利等各个方面拥有平等的权利和待遇。促进教育公平，将进城务工人员随迁子女义务教育纳入教育发展规划和财政保障范畴，免费享受中等职业教育和普惠性学前教育，制定实施随迁子女在市区参加升学考试的具体办法。实施就业培训工程，完善农业转移人口职业技能认定体系，鼓励高等院校、职业院校和培训机构开展职业教育和技能培训。优化城镇卫生计生资源配置，推进城乡保障制度衔接，加快农业转移人口纳入社区卫生计生服务体系管理和社会保险覆盖范围，实现农业转移人口与城镇居民社会保障同步。建立公平效率兼顾的住房保障体系，合理建构住房供给结构，采取公共租赁、购房补贴等方式，改善进城务工者的居住条件。督促企业与进城务工人员建立稳定的劳资关系、合同关系，依法参加养老、医疗、工伤、失业、生育等社会保险，切实维护进城务工人员的劳动所得和合法权益。

（七）坚持绿色发展，着力推动生态文明建设迈上新台阶。

良好生态环境是最普惠的民生福祉，要推动环境保护与经济发展相生共融，大力发展生态经济，保护好青山绿水和蓝天净土。

推进生态项目建设。着力推动国家循环经济示范城市建设，加快构建循环型工业、农业、服务业、社会“四位一体”体系，全面推行低碳、环保、绿色、节约的生产生活方式。

加快建设国家城市矿产示范基地，重点以产业园区为平台，着力构建再生金属、再生塑料等循环产业链，提升区域循环经济发展水平和综合竞争力。

加快推进城市可再生能源建筑应用推广示范工作。

大力发展生态产业，推进健康养生、生态农业、休闲旅游等重点项目建设，培育新的经济增长点。

完成节能减排任务。严控高耗能、高污染项目，严格节能评估，坚决淘汰落后产能，加快推进清洁生产。引导企业开展节能低碳行动，实施一批节能重点工程和资源节约、循环利用示范项目。认真落实减排工作责任和环保“一岗双责”制度，重点抓好市第三污水处理厂等污水处理设施及配套管网建设，加强重点水源保护区排污口治理，推进规模化养殖场减排、机动车减排等工作，完成“十二五”节能减排任务。全面开展核发机动车环保合格标志工作，完成黄标车及老旧车辆淘汰任务。

加强生态保护。严格执行新环保法，加大污染防治、环境整治、环保执法力度。加快建设梧州市静脉产业园。加强西江、浔江、桂江三大流域森林和水环境保护，推进重金属污染、土壤污染等专项治理工程。抓好广西雨水收集利用建设试点城市工作，加强城市地下管网建设改造和规范治理。全力推进“美丽梧州·生态乡村”建设，扎实开展“村屯绿化”、“饮水净化”和“道路硬化”专项活动，抓好田园废弃物回收处理工作，推广和应用清洁农业生产技术，建设富裕文明、生态美好的新农村。“山清水秀生态美”是梧州最宝贵的财富，绿水青山就是金山银山。我们要以更大的决心和气魄，保护生态，治理环境，让这笔财富永续利用、代代相传！

（八）大力改善民生，着力推动和谐社会建设取得新进步。

改善民生，始终是政府工作的出发点和落脚点。要坚持以问题为导向，多谋民生之利，多解民生之忧，确保人民安居乐业、社会和谐稳定。

加强就业和社会保障工作。落实更积极的就业创业政策，统筹做好高校毕业生、城镇就业困难人员、农村转移人口就业工作，全年实现城乡新增就业7.5万人次，开展各类培训4万人。

抓好住房保障，建设保障性住房3471套。

完善和落实各项社会保险政策，实施全民参保登记计划，推进基本公共服务全覆盖。

全面推行职工基本医疗保险制度和新农合制度市级统筹，将城乡居民医疗保险补助标准从320元提高到380元。

探索医养融合新模式，积极推进居家养老，完善养老服务体系。

全面开展临时救助工作，编密织牢基本民生保障网。

打造梧州教育升级版。进一步加大对教育投入，继续抓好教育发展十大重点工程，重点抓好农村新建、改扩建幼儿园建设，推进农村义务教育学校、农村义务教育薄弱学校改造工程和市第一中学新校园、梧州高中新校园二期工程等项目。

加大义务教育均衡发展力度，推动高中走多样化、特色化、内涵式发展道路。

深化职业教育管理体制改革，抓好全市职业教育发展统筹，加强重点学科、专业建设，加快微软IT学院建设，推进政校企合作，促进产教研融合，构建现代职业教育体系，提升职业教育服务地方经济社会发展水平。

支持梧州学院推进综合改革，加快转型发展。

发展卫生计生文体事业。深化医药卫生体制改革，推进县级公立医院改革，完成市人民医院新院搬迁工作，加快推进工人医院扩建、岭南中医药研究所、妇幼保健院业务楼扩建等项目建设，筹建苍梧县中医院、妇幼保健院，启动粤桂人民医院筹建和市红十字会医院扩建工作。大力推进智慧医疗建设，开展全国基层中医药先进单位创建工作。做好重大疾病防控工作。坚持计划生育基本国策，落实各项计生惠民政策。深化文化体制改革，激发文化活力。统筹推进城乡文化设施建设，谋划启动现代化书城项目，确保市文化大厦、市新图书馆上半年投入使用，建设62个村级公共服务中心，推进公共文化基础设施场所免费开放，促进基本公共文化服务均等化。挖掘历史文化内涵，进一步加强文物保护及非物质文化遗产保护、传承与展示，开展“国家历史文化名城”申报工作。做好广西第十三届运动会各项筹备和组织工作，当好东道主，努力办成一届文明、精彩的体育文化盛会。

加强和创新社会治理。推进法治社会建设，加快公共法律服务体系建设，完成“六五”普法任务。

加强社区、乡村基层组织建设，严格落实维稳工作责任制，抓好社会稳定风险评估和矛盾纠纷排查调处工作，解决好土地征用、房屋拆迁、环境污染治理过程中引发的矛盾。

深入推进平安梧州建设，强化社区网格化管理，构建立体化治安防控体系，提升群众安全感和满意度。

创新信访体制机制，不断提升信访工作整体水平。

严格落实“党政同责、一岗双责”制度，切实加强安全生产工作。

加强食品药品安全治理体系和治理能力建设，大力整治“餐桌污染”，严厉打击生猪私屠滥宰行为，不断提升安全保障水平。

进一步强化应急管理工作，提高预防和应对突发公共事件能力。

大力实施精准扶贫。深入推进新一轮扶贫开发攻坚，首批筹措资金7300万元以上，以3个扶贫开发工作重点县、179个贫困村为主战场，大力改善贫困村基础设施，促进贫困村产业发展，推进扶贫开发与农村社会保障相衔接，增强贫困村发展后劲。抓好库区移民工作，大力改善移民生产生活条件，增强移民自我发展能力。

办好民生实事工程。筹措资金43亿元以上，继续实施12项为民办实事工程，重点推进一批农村中小学危旧校舍和义务教育薄弱学校改造工程，新建一批普惠性幼儿园，硬化 380条村屯道路，改造3900户农村危房，新建1000座农村沼气池，解决好12万农村人口饮水安全问题，实施市区小街小巷和排水设施改造工程等。

各位代表，民生无小事，保障和改善民生没有终点站。我们要把群众当家人看，把民生当家事办，在为民办实事上，坚决做到不失真、不走样，让人民生活年年都有新变化，一年更比一年好！

（九）推进依法行政，着力推动法治政府建设开创新局面。

深入贯彻落实党的十八届四中全会精神，全面推进依法行政，加快建设法治政府，保障人民赋予的权力始终为人民谋利益。

依法履行政府职能。全面履行经济调节、市场监管、社会管理、公共服务、环境保护等职责。市县两级全面推行政府权力清单、责任清单和负面清单制度，深化行政审批制度改革，精简行政审批事项，把管理事务从事前审批更多地转移到事中、事后监管。大力转变工作作风，对法定职责范围内的行政事务，要以抓铁有痕、踏石留印的精神狠抓工作落实，坚决纠正不作为、乱作为，坚决惩处失职、渎职。

坚持依法决策和行政。完善重大事项公众参与、专家论证、风险评估、合法性审查、集体决策等制度，落实领导干部决策责任追究制度，促进决策科学化、民主化、法治化。全面建立政府法律顾问制度。加强市县两级政府行政执法改革和管理，理顺行政执法体制，合理配置执法力量。推行行政执法权力清单制度，健全完善执法依据、程序公开制度。严格施行行政执法人员资格管理。全面落实行政执法责任制，健全依法维权和化解矛盾纠纷机制，加大关系群众切身利益的重点领域执法力度，进一步规范行政执法行为。

强化权力监督制约。自觉接受人大监督，主动接受人民政协的民主监督，认真办理人大代表议案、建议和政协提案，广泛听取民主党派、工商联、无党派人士和人民团体意见。实施行政执法专项监督检查制度，强化对重点行业、重点领域行政执法的监督。强化政府内部层级监督和行政应诉工作，实行纠错问责机制，健全问责方式和程序，严肃追究违纪违法行政工作人员的责任。推进政务公开，依据权力清单，向社会全面公开政府职能、法律依据、实施主体、职责权限、管理流程、监督方式等事项，加快建设阳光政府。

加大作风建设和反腐倡廉力度。巩固和扩大党的群众路线教育实践活动成果，继续抓好整改落实和建章立制，严防“四风”反弹，营造风清气正的发展环境。严格落实“八项规定”要求，加强“三公”经费管理，降低行政运行成本。严格落实党风廉政建设责任制，坚持“一岗双责、守土有责”，加强惩治和预防腐败体系建设，加强行政监察和审计监督，强化重点领域和关键环节的权力监管，健全用人、用钱、用地、用权制度，把权力关进制度的笼子里。

同时，我们还要加强国防教育和国防后备力量建设，推动军民融合深度发展，大力开展双拥共建活动，提高军转安置和优抚工作水平。依法保障公民特别是妇女、未成年人和残疾人的合法权益。支持工会、共青团、妇联等人民团体发挥桥梁纽带作用，继续做好新闻出版广电、民族宗教、外事侨务、统计、人民防空、气象、地震、水文、邮政、通信、地方志、档案等工作。切实做好“十二五”终期评估，高质量编制“十三五”规划。

各位代表！时代创造机遇，奋斗开创未来。让我们紧密团结在以习近平同志为总书记的党中央周围，在自治区党委、政府和市委的坚强领导下，紧紧依靠全市干部群众，以更加饱满的精神，更加昂扬的斗志，更加务实的作风，抢抓机遇，扎实工作，奋勇前行，为加快建设西江黄金水道上向东开放的龙头城市，全面建成小康社会而努力奋斗！
